# Primary ectopic atypical meningioma in the renal hilum: a case report

**DOI:** 10.1186/1471-2407-14-763

**Published:** 2014-10-14

**Authors:** Ye-qing Mao, Wei Zhang, Wen-juan Yin, Shao-xing Zhu

**Affiliations:** Department of Urology, The First Affiliated Hospital of Zhejiang University, Hangzhou, China, No.79 qingchun Road, Shangcheng District of Hangzhou, Zhejiang Province Hangzhou, China; Department of Urology, Zhejiang Cancer Hospital, Hangzhou, China, No.38 guangji Road, Gongshu, District of Hangzhou, Hangzhou, Zhejiang Province China; Department of Pathology, Zhejiang Cancer Hospital, Hangzhou, China, No.38 guangji Road, Gongshu District of Hangzhou. Hangzhou, Zhejiang Province China

**Keywords:** Ectopic meningioma, Atypical meningioma, Retroperitoneal mass, Renal hilum

## Abstract

**Background:**

Primary ectopic atypical meningioma involving the renal hilum is rare. This is, to our knowledge, only the second case report of a primary retroperitoneal meningioma and the first case of an atypical subtype in this location.

**Case presentation:**

A 53-year-old Han Chinese man presented with a 2-year history of left-side flank pain. An oval-shaped retroperitoneal mass was found in the left renal hilum on computed tomography, which was resected *en bloc* along with the kidney via laparotomy. According to the World Health Organization criteria, the tumor was histopathologically classified as a meningioma (Grade II, atypical). Five years later, the tumor recurred at the primary site with a similar histopathology. The patient received palliative resection, followed by radiotherapy (4500 cGy in 25 fractions). No relapse was found at 6-month follow-up.

**Conclusion:**

We describe the clinical, radiographic and histopathological features of an unusual case of aggressive ectopic meningioma in the renal hilum. The patient presented with a massive retroperitoneal tumor without primary cerebral or secondary metastatic lesions; the preoperative diagnosis was naturally confined to the common retroperitoneal malignancies. This case is of interest to oncologists, because of both its rare location and aggressiveness; it not only enriched the spectrum of primary ectopic meningioma, but also reminded us of potential recurrence of an atypical meningioma. This case raises the issue of the etiology of such a rare tumor that needs further investigation, and more importantly demands long-term follow-up result.

## Background

Meningioma is a central nervous system (CNS) tumor arising from arachnoidal or meningothelial cells [1]. It is one of the most frequently diagnosed primary brain tumors, comprising approximately one-third of CNS tumors in adults in the United States [1]. Meningioma is normally of slow-growing type, with the aggressive lesion accounting for <10% of cases [1].

Despite being a common intracranial tumor, meningioma has been reported to originate from unexpected sites such as bone, scalp, paranasal sinuses, parotid gland, parapharyngeal space, mediastinum, lung, and even the abdomen [1, 2]. Ectopic meningiomas are empirically defined as primary tumors located in sites without arachnoidal cells, ruling out local extension or metastasis. In 1996, Huszar et al. reported the first case of primary ectopic meningioma in the retroperitoneum [2]. Here, we present a case of primary meningioma in the renal hilum with atypical presentation.

## Case presentation

**Figure 1:**
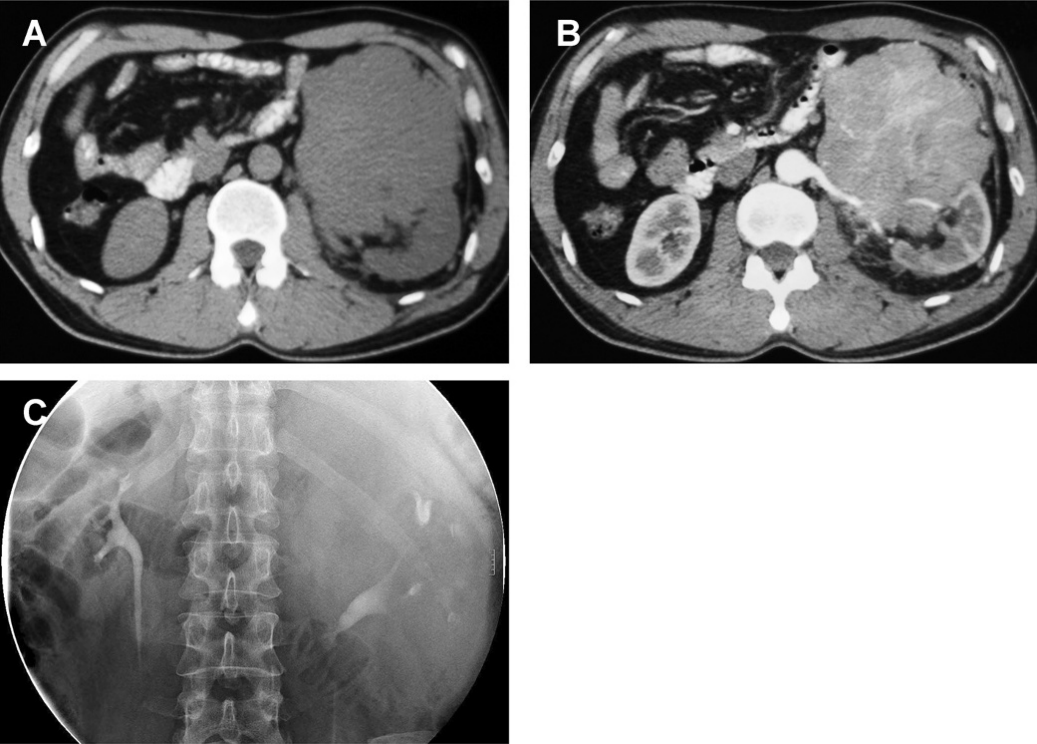
**CT scanning and intravenous urography show the mass in the left renal hilum.** The tumor displaced tissue near the renal hilum, and invaded the parenchyma and renal vessels. **A**: The plain scanning of the tumor. **B**: The contrast-enhanced phase of imaging. **C**: The collecting system of the affected kidney was compressed and distorted.

A 53-year-old man attended our clinic with a 2-year history of left-side, dull flank pain. The progressive pain was aggravated by being in the recumbent position, without groin-radiating pain. No emaciation, fever, hematuria or CNS symptoms were present. Physical examination revealed a large solid mass with an impalpable border in the left upper quadrant of the abdomen. Abdominal computed tomography (CT) scanning confirmed an irregular mass with soft-tissue density near the left renal hilum, invading the parenchyma. On imaging, the bulky mass had transient heterogeneous enhancement, which was more prominent in the center. There was no lymphadenopathy or metastasis (Figure 1A and B). Intravenous urography demonstrated that the enlarged left collecting system was pushed downward by the tumor, with collapsed and lengthened calices; however, there were no visible filling defects (Figure 1C). The routine laboratory tests were unremarkable. The patient was initially diagnosed with primary renal cancer.

During the operation, a well-defined, oval tumor measuring 10.9 × 8.5 × 17.5 cm was found in the left renal hilum. It infiltrated the renal parenchyma, encased the renal vessels, and significantly displaced the pancreas and splenic vessels. However, there were no enlarged lymph nodes in the retroperitoneum. Radical nephrectomy was performed, and the tumor was resected *en bloc* without violation of the capsule. Frozen sections showed that the tumor was renal spindle cell carcinoma. The operation was not eventful and the patient was timely discharged.

**Figure 2:**
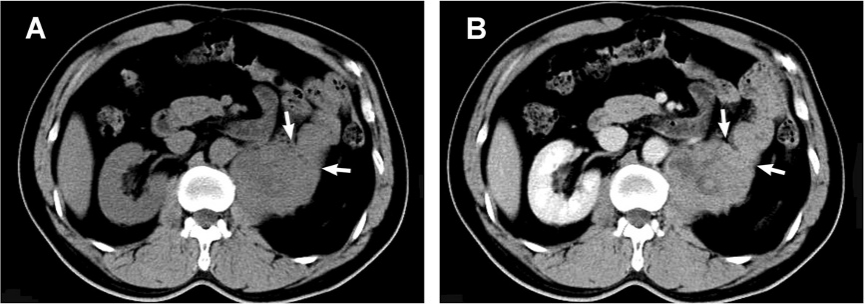
**Recurrent mass in the renal fossa. A**: CT scanning demonstrates that the tumor attached closely to the psoas muscle and invaded the jejunum (arrow). **B**: delayed, mild enhancement was prominent in the venous phase.

He was periodically followed up in our clinic. Multiple CT scans of the abdomen were performed, with the most recent being 2 years postoperatively. Five years after surgery, an asymptomatic recurrent lesion was identified attached to the psoas muscle and jejunum in the left renal fossa. The patient was asymptomatic. On CT scan, the tumor was shown to have delayed moderate enhancement, which implies underdeveloped vasculature (Figure 2). Doppler ultrasonography revealed moderate blood flow within the hypoechoic mass (data not shown). To identify other potential lesions, we performed craniocerebral and thoracic CT scanning, which revealed no mass lesion, displacement or bone destruction in the area.

The patient was readmitted for surgery. Intraoperatively, we found an 8.0 × 7.5-cm mass firmly attached to the left psoas muscle, with infiltration to the proximal jejunum. We then performed a debulking operation with resection of 10 cm of the involved proximal jejunum.

**Figure 3:**
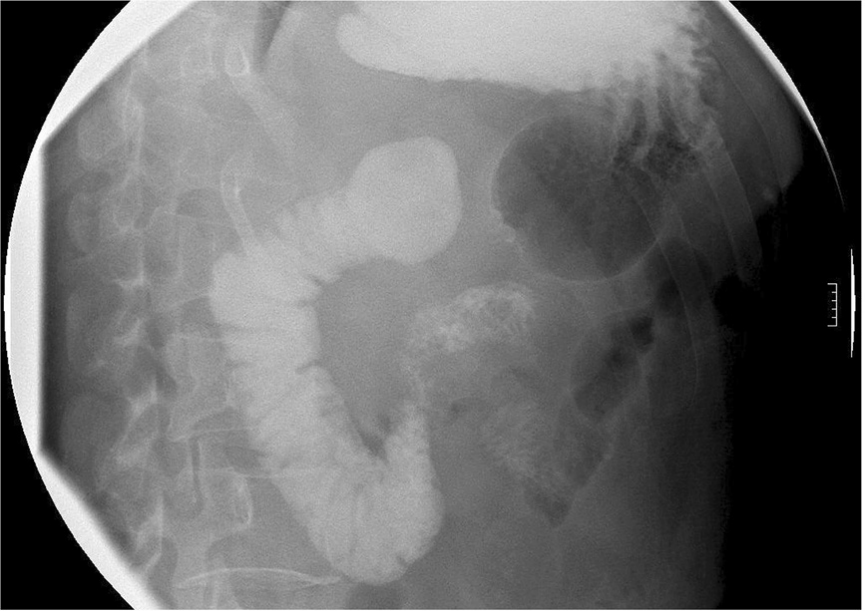
**Images of small bowl obstruction.** Gastrointestinal imaging showed a suspected obstruction at beginning of the jejunum.

The second operation was complicated by sudden onset of persistent abdominal pain, nausea and vomiting at postoperative day 7. Physical examination showed upper abdominal distention without tenderness; the vital signs were stable. Gastrointestinal imaging revealed small bowel obstruction, presumably because of anastomotic stricture (Figure 3). We placed a nasogastric tube and later started total parenteral nutrition. After rehabilitation, the patient was transferred to the medical oncology ward and received local external radiotherapy (4500 cGy in 25 fractions). No recurrence has been identified on abdominal imaging 6 months after the second operation.

**Figure 4:**
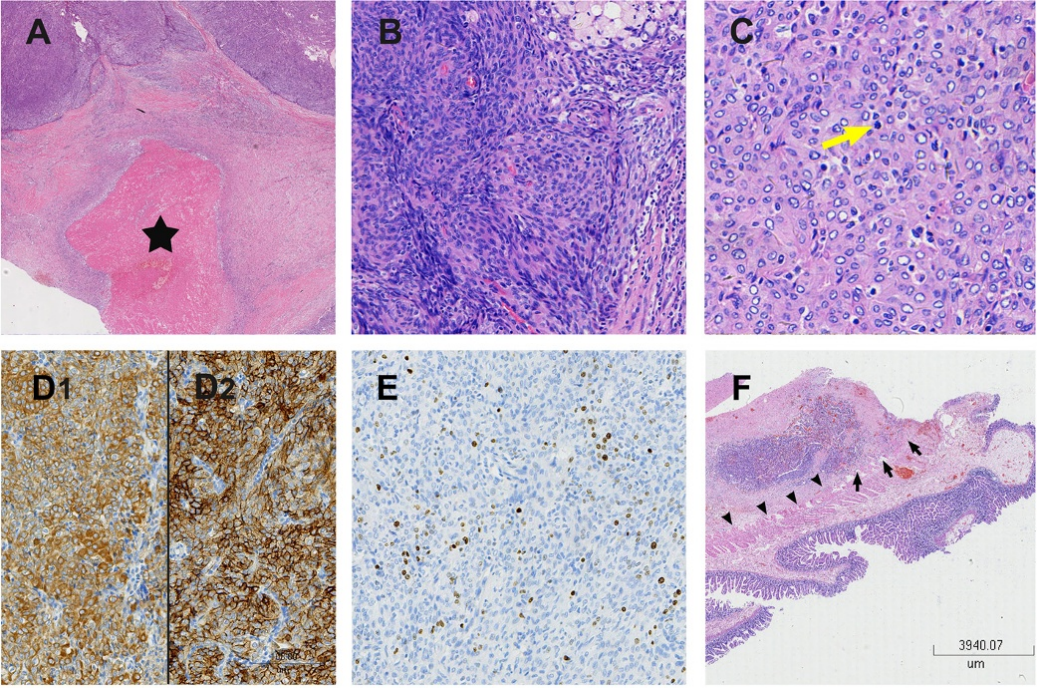
**Photomicrographs of histopathological examination. A**: Necrotic foci appeared as an area filled with eosinophilic amorphous material (pentagram) [hematoxylin–eosin (HE)-stained, original magnification × 4]. **B**: Epithelioid spindle-shaped cells form in a storiform and sheet-like growth pattern (HE-stained, original magnification × 20). **C**: Fine structure of the tumor cells, and especially a typical mitotic figure (yellow arrow) are clearly demonstrated (HE-stained, original magnification × 40). **D1**: Immunohistochemical staining of EMA was positive (original magnification × 20). **D2**: Immunohistochemical staining of vimentin was positive (original magnification × 20). **E**: The Ki67 was estimated at 15%. **F**: Infiltration of the tumor (arrow) into longitudinal intestinal muscle layer (arrowhead; HE-stained, original magnification × 4).

Microscopically, both specimens showed features of multifocal necrosis (Figure 4A). Evaluation under a higher magnification showed significant hypercellularity, with epithelioid tumor cells arranged in a whorl or sheet-like pattern (Figure 4B). These polygonal cells were characterized by abundant cytoplasm, vacuolated nuclei with fine chromatin and small nucleoli; the mitotic figures were evident and were estimated to be 5–8 mitotic cells per 10 high-power fields (Figure 4C). Immunohistochemically, the tumor cells were extensively positive for epithelial membrane antigen (EMA) and vimentin (Figure 4D1 and D2), and locally for total cytokeratin (CK). The remaining staining profiles were almost negative, including S-100, HMB-45, bcl-2, melan-A, myelin basic protein (MBP), neurofilament (NF), chromogranin A (CgA), C-kit, glial fibrillary acidic protein, Glut1, CD21, CD23, CD31, CD34, CD35, CD117, WT-1, smooth muscle actin, actin, inhibin-α, Syn and progesterone receptor (PR). The cell proliferative index marker Ki67 was estimated at 15% (Figure 4E). The histopathological diagnoses of both tumors were atypical meningiomas [the World Health Organization (WHO) 2007, Grade II], with the recurrent lesion invading the intestinal tissue (Figure 4F). Given that no intracranial or intraspinal lesion was identified, the patient was finally diagnosed with primary ectopic atypical meningioma.

### Discussion

The histological origin of ectopic meningioma is still elusive. Some authors believe that the tumors might derive from ectopic, misplaced, or migrating arachnoid cells [3–5]. A recent analysis of oncogenesis have suggested a different histogenesis between ectopic and CNS lesions [6]. Some investigators have demonstrated that the tumor may originate from perineurial cells or pluripotent mesenchymal cells [5]. Therefore, we speculate that the meningioma in our case might have arisen from a nest of trapped arachnoid cells in the retroperitoneum during embryogenesis, or more likely from nerve sheath cells, given the existing reports of renal schwannoma involving the renal hilum [7].

An accurate preoperative diagnosis of ectopic meningioma can be challenging. Despite radiographic similarities between our case and aggressive intracranial meningioma on retrospective review, including lobulation, cyst formation, hemorrhage, necrosis, local invasion and heterogeneity with brisk enhancement [8], there is a long list of differential diagnosis of retroperitoneal tumors.

A preoperative differential diagnosis of benign and malignant tumors at and around the renal bed can be difficult. The topographic location of the tumor in our case is reminiscent of renal cell cancer (RCC). As the largest subtype, clear-cell RCC is a heterogeneous regular mass with destruction of the parenchyma and kidney contours, and it shows strong contrast enhancement and rapid attenuation on CT. Papillary and chromophobic RCCs tend to be homogeneous and undergo mild enhancement without frequent necrosis. After a RCC invades the renal pelvis, it is often accompanied by hematuria. Large RCCs often portend nodal disease, extension into vessels, and distant metastasis.

Epithelioid angiomyolipoma, an unusual subtype of renal angiomyolipoma, has been recently identified as a potential malignancy. These non-fatty lesions invariably appear as heterogeneous soft-tissue masses on CT; some giant angiomyolipomas even exhibit local invasiveness and lymph node metastasis. Enlarged vessels, hemorrhage, and spontaneous kidney rupture are helpful for diagnosis if identified [9].

Leiomyosarcomas are arguably the most common retroperitoneal sarcomas. These hypervascular malignancies, mainly originating from vessel smooth muscle cells, are more frequently located in the uterine or gastrointestinal tracts. They appear as poorly circumscribed, lobulated masses with iso/hypodensity and delayed circumferential contrast enhancement on CT. They may also have extensive necrosis, cystic degeneration, and hemorrhage. Imaging findings of compression, invasion, or obstruction of the vena cava could be regarded as a characteristic of leiomyosarcoma [10].

Liposarcomas, another group of common sarcomas of the retroperitoneum, are considered relatively easy to recognize. However, the rare pleomorphic liposarcoma may pose a challenge for differentiation because of a non-fatty appearance. Extensive necrosis, hemorrhage and metastasis are inadequate to discriminate them from other soft-tissue malignancies [11].

Considering the non-pathognomonic imaging presentations of this case and unexpectedly overlap in imaging features of many retroperitoneal tumors, pathological analysis through core biopsy is still a determinate diagnostic tool.

According to the WHO 2007 standards, meningioma is histologically classified into three types: predominant Grade I, benign tumors; and rare Grade II and III, aggressive tumors. Histological Grade II meningioma, also termed atypical meningioma, is estimated to account for 4.7–7.2% of the entity [12]. It is diagnosed when four or more mitotic cells per 10 high-power fields and/or three or more of the following: increased cellularity, small cells, necrosis, prominent nucleoli, sheeting growth, and/or brain invasion in an otherwise Grade I tumor [12].

Although the pathological guidelines are straightforward, the criterion resembles a series of carcinomas and sarcomas that need differentiation. In this case, the tumor should be distinguished from several retroperitoneal masses by means of immunohistochemistry and fluorescence in situ hybridization. The biphasic subtype of synovial sarcoma exhibits epithelioid and mesenchymal differentiation, typically supported by positive staining of EMA, vimentin, and CK. However, the cells grow in nested acinar patterns, and more importantly, the characteristic SYT-SSX fusion gene is diagnostic [13, 14], which was negative in our case (data not shown). Epithelioid variant of hemangiopericytoma may also present with positive staining of EMA and vimentin, but positive bcl-2 and CD34 are helpful for differentiation [15, 16]. Soft-tissue perineurioma, a rare subtype not arising from an identifiable nerve, is found mainly in the superficial layers of the limbs and trunk; its typical profile is strong positive staining of EMA and CD34 [17].

The microscopic presentation and clinical features of the tumor in our case were in line with the WHO 2007 Meningioma Classification (Grade II), and it could be distinguished from the differential candidates above. Despite a controversial marker to predict tumor prognosis, the relatively high level of Ki67 did indicate later recurrence in our case [18]. In addition, it is noteworthy that the PR staining in our case was negative. PR staining positivity has been reported to appear mainly in classic meningioma with good prognosis. Therefore, the negative staining might suggest tumor aggressiveness [19].

The only definitive treatment for meningioma is curative resection. However, no successful precedent could be found for treating retroperitoneal meningioma. In the case reported by Huszar et al., the patient relapsed 6 months after surgical resection and survived for only 2 years under chemotherapy [2]. Our case was milder, but it still demonstrates certain aggressiveness including infiltration into the muscle and intestine, and more extensive positive markers (EMA and vimentin) and necrosis. Finally, the patient was recommended to receive radiotherapy, the standard adjuvant therapy for inoperable or recurrence-prone intracranial meningioma [20]. The outcome remains to be seen.

## Conclusion

To the best of our knowledge, this is only the second case report of a primary retroperitoneal meningioma and the first case of an atypical subtype in this location. Like the previously reported one, our case presented with invasiveness and gross volume, which warranted regular examination and timely treatment. Considering its rarity and non-pathognomonic presentation, the best method of preoperative diagnosis might be core biopsy. Surgical resection combined with adjuvant radiotherapy may be an alternative treatment, but urgently needs assessment.

## Consent

Written informed consent was obtained from the patient for publication of this Case report and any accompanying images. A copy of the written consent is available for review by the Editor of this journal.

## References

[1] Claus EB, Morrison AL, DeMonte F, McDermott MW, Al-Mefty O (2011). Epidemiology of Meningiomas. Al-Mefty’s Meningioma.

[2] Huszar M, Fanburg JC, Dickersin GR, Kirshner JJ, Rosenberg AE (1996). Retroperitoneal malignant meningioma: A light microscopic, immunohistochemical, and ultrastructural study. Am J Surg Pathol.

[3] Ishigaki D, Arai H, Sasoh M, Ogasawara K, Uesugi N, Sugai T, Nakamura S, Ogawa A (2007). Meningioma in the posterior fossa without dural attachment. Neurol Med Chir.

[4] Llauger J, Aixut S, Canete N, Palmer J, Sola M, Bague S (2008). Meningioma of the scapula. Skeletal Radiol.

[5] Lockett L, Chiang V, Scully N (1997). Primary pulmonary meningioma: report of a case and review of the literature. Am J Surg Pathol.

[6] Liu Y, Wang C, Zhu S, Li F, Wang H, Liu M, Zhang L, Wu C (2011). Clinical characteristics and treatment of ectopic meningiomas. J Neurooncol.

[7] Hung SF, Chung SD, Lai MK, Chueh SC, Yu HJ (2008). Renal Schwannoma: case report and literature review. Urology.

[8] Thurnher MM, Osborn AG, Salzman KL, Barkovich AJ (2009). Anatomy-based diagnosis: Skull, Scalp and Me ninges Neoplasms. Diagnostic Imaging: Brain.

[9] Froemming AT, Boland J, Cheville J, Takahashi N, Kawashima A (2013). Renal epithelioid angiomyolipoma: imaging characteristics in nine cases with radiologic-pathologic correlation and review of the literature. AJR Am J Roentgenol.

[10] O’Sullivan PJ, Harris AC, Munk PL (2008). Radiological imaging features of non-uterine leiomyosarcoma. Br J Radiol.

[11] Kumarasamy NA, Gayer G (2011). Retroperitoneal sarcomas. Semin Ultrasound CT MR.

[12] Perry A, Louis DN, Ohgaki H, Wiestler OD, Cavenee WK (2007). Meningiomas. World Health Organization Classification of Tumours of the Central Nervous System.

[13] Weiss SW, Goldblum JR, Weiss SW, Goldblum JR (2001). Malignant soft tissue tumors of uncertain type. Enzinger and Weiss’s Soft Tissue Tumors.

[14] Fisher C, De Bruijin DHR, Geurts Van Kessel A, Fletcher CDM, Unni KK, Mertens F (2002). Synovial sarcoma. Pathology and Genetics. Tumours of Soft Tissue and Bone.

[15] Rajaram V, Brat DJ, Perry A (2004). Anaplastic meningioma versus meningeal hemangiopericytoma: immunohistochemical and genetic markers. Hum Pathol.

[16] Commins DL, Atkinson RD, Burnett ME (2007). Review of meningioma histopathology. Neurosurg Focus.

[17] Hornick JL, Fletcher CD (2005). Soft tissue perineurioma: clinicopathologic analysis of 81 cases including those with atypical histologic features. Am J Surg Pathol.

[18] Roser F, Samii M, Ostertag H, Bellinzona M (2004). The Ki-67 proliferation antigen in meningiomas. Experience in 600 cases. Acta Neurochir.

[19] Roser F, Nakamura M, Bellinzona M, Rosahl SK, Ostertag H, Samii M (2004). The prognostic value of progesterone receptor status in meningiomas. J Clin Pathol.

[20] Combs SE, Schulz-Ertner D, Debus J, von Deimling A, Hartmann C (2011). Improved correlation of the neuropathologic classification according to adapted world health organization classification and outcome after radiotherapy in patients with atypical and anaplastic meningiomas. Int J Radiat Oncol Biol Phys.

[CR21] The pre-publication history for this paper can be accessed here:http://www.biomedcentral.com/1471-2407/14/763/prepub

